# Longer mtDNA Fragments Provide a Better Insight into the Genetic Diversity of the Sycamore Lace Bug, *Corythucha ciliata* (Say, 1832) (Tingidae, Hemiptera), Both in Its Native and Invaded Areas

**DOI:** 10.3390/insects13020123

**Published:** 2022-01-25

**Authors:** Ferenc Lakatos, Katalin Tuba, Boglárka Bender, Hisashi Kajimura, Viktória Tóth

**Affiliations:** 1 Faculty of Forestry, Institute of Forest and Natural Resource Management, University of Sopron Bajcsy-Zsilinszky u. 4, H–9400 Sopron, Hungary; tuba.katalin@uni-sopron.hu (K.T.); bender.bogi.amelie@gmail.com (B.B.); toth.viktoria@uni-sopron.hu (V.T.); 2 Laboratory of Forest Protection, Graduate School of Bioagricultural Sciences, Nagoya University, Chikusa, Nagoya 464-8601, Japan; kajimura@agr.nagoya-u.ac.jp

**Keywords:** *Corythucha ciliata*, mtDNA, COI fragment length, population genetics, phylogeny, invasive insect

## Abstract

**Simple Summary:**

The sycamore lace bug (*Corythucha ciliata* Say, 1832) is one of the most abundant and widespread pests on plane trees (*Platanus* spp.) across the globe. The native range of the species is in North America, but it has been introduced to Europe (1964), South America (1985), Asia (1995), Australia (2006), and Africa (2014). To understand the genetic background behind this successful colonisation, we analysed a fragment (1356 bp) of the mitochondrial DNA. The 327 individuals revealed 17 haplotypes forming two separated groups. One group includes North American and Japanese individuals, while the other group comprises North American, European, and Asian individuals. We found a much higher genetic diversity in the native area (North America, 12 haplotypes) than in the invaded areas (Europe, five and Asia, four). The longer DNA fragment provided detailed information about the genetic structure of the species both in its native range and in the invaded areas, but the shorter DNA fragment could not provide a clear link between the genetic variation and the geographic origin.

**Abstract:**

The sycamore lace bug (*Corythucha ciliata* Say, 1832) is of North American origin, but after its introduction to Europe (1964), South America (1985), Asia (1995), Australia (2006), and Africa (2014), it became an abundant and widespread pest on plane (*Platanus* spp.) trees. We analysed a 1356 bp long fragment of the mtDNA (COI gene) of 327 sycamore lace bug individuals from 38 geographic locations from Europe, Asia, and North America. Seventeen haplotypes (17 HTs) were detected. *C. ciliata* populations from North America exhibited higher haplotype diversity (12 HTs) than populations from Europe (6 HTs), Asia (4 HTs), or Japan (2 HTs). The haplotypes formed two haplogroups separated by at least seven mutation steps. One of these mutation steps includes HTs from North America and Japan. Another includes HTs from North America, Europe, and Asia. Haplotypes from Asia Minor, the Caucasus, and Central Asia are linked to haplotypes from Europe, while haplotypes found in Japan are linked to haplotypes found in North America only. The incorporation of published data from the GenBank into our dataset (altogether 517 individuals from 57 locations, but only 546 bp long fragment of the mtDNA) did not show any structure according to the geographic origin of the individuals.

## 1. Introduction

Understanding the success of an invasive insect species requires knowing the source and pathway(s) of invasion and the spatial distribution of intraspecific diversity [1,2]. Without that knowledge, establishing efficient control measures proves difficult. Several studies conducted in recent decades have investigated various genetic markers to address this concern [3,4,5,6,7].

The native range of the sycamore lace bug (SLB) (*Corythucha ciliata* Say, 1832) is in North America, where its main hosts are *Platanus* spp. [8]. The invasion history of sycamore lace bug across Europe is well documented. The first record was in Italy (1964), followed by a high-speed spread across the continent: 1970, Croatia (ex-Yugoslavia, Zagreb); 1972, Slovenia (Ljubljana); 1973, Serbia; 1975, South France and Switzerland; 1976, Hungary; 1978, Spain; 1982, Austria; 1983, Germany and Czechoslovakia; 1987, Bulgaria; 1990, Romania; 1994, Portugal; 1988, Greece; 1996, Russia; 2005, Moldova; 2006, United Kingdom and Belgium; 2007, Turkey and Ukraine; 2008, Netherlands and Georgia; 2009, Poland; 2011, Macedonia; and 2012, Luxemburg. No continent has remained unaffected: South America, Chile (1985); Asia, South Korea (1995), China (2002), Japan (2003), and Uzbekistan (2017); Australia (2006); and Africa, South Africa (2014) [9,10,11,12,13,14,15,16,17,18,19,20,21,22,23,24,25,26,27,28,29,30,31,32,33,34,35,36,37,38,39,40,41,42,43,44]. *C. ciliata* has spread via anemochore and antropochore transportation [10,11,12,17,22,45,46].

Plane trees (taxon names noted according to the Catalogue of Life [47]; *Platanus occidentalis* L., *P. orientalis* L., and *P. hybrida* Brot.), are major host plants [8,48] of *C. ciliata,* but feeding has also been recorded on *Fraxinus* sp., *Morus alba* L., *Broussonetia papyrifera (L.) Vent.*, *Carya ovata* (P. Mill.) K. Koch, and *Chamaedaphne* sp. [8]. However, another publication [48] reports other possible host plant species as well (*Quercus laurifolia* L., *Liquidambar sytraciflua* Michx., and *Euphorbia pulcherrima* Willd. Ex Klotzsch), but their list does not include *Broussonetia*, *Carya*, *Chamaedaphne*, or *Fraxinus* as hosts. Torres-Miller [49] only detected it on *P. occidentalis* from West Virginia.

In North America, heavy infestations and damages were reported on ornamental and shade plane trees, especially in the eastern part of the United States [50,51,52,53]. Coyle et al. [54] investigated the sycamore lace bug because *P. occidentalis* is an important tree species in the managed hardwood forests of North America. They concluded that the species does not cause considerable damage under traditional forest conditions. The sycamore lace bug was found mainly on *Platanus* species in the invaded areas [11,21,22,41,45,46,55,56]. However, nymphs and adults were also detected on maples (*Acer*) and ash trees (*Fraxinus*) from Georgia [18]. Sycamore lace bug individuals cause aesthetic damage by sucking sap from the leaves, but they may play a role as a vector of various diseases as well (e.g., *Erysiphe platani* (Howe) U. Braun & S. Takam., 2000). Plane trees are widely used as ornamental tree species in the northern hemisphere [57,58,59,60].

Imagos overwinter under tree bark. The sycamore lace bug is well adapted to the extreme cold temperatures (−30 °C) [21], but adults can tolerate extreme high temperatures (35–41 °C) as well, [61,62] which allows the species a wide potential distribution area. Ju et al. [63] revealed phenotypic synchronicity between SLB individuals and their host.

Various methods to control SLB have been investigated. Yoon et al. [20]) examined the effect of bistrifluron (chitin synthesis inhibitor), and Pavela et al. [64] applied azadirachtin (active ingredient of the neem tree *Azadirachta indica* A. Juss.) with good results. Several studies assessed the natural enemy complex of SLB [65]. Entomopathogenic fungi (e.g., *Beauveria bassiana* [Bals.-Criv.] Vuill., 1912), nematodes (e.g., *Steinernema* sp., *S. carpocapsae* [Weiser, 1955]) and generalist predatory insects (e.g., *Chrysoperla lucasina* [Lacroix, 1912] [Neuroptera: Chrysopidae]) proved to be possible biological control agents [66,67,68].

A few genetic studies have been conducted on *C. ciliata* in the last decade. One single individual was analysed with microsatellites markers from China [69]. The gene expression profiles were studied [14,62,70] as a part of a DNA barcode library construction project [71,72]. Kocher et al. [73] published a whole mitogenome compared with the avocado lace bug (*Pseudacysta perseae*, Heidemann 1908) using a genome skimming approach. Yang et al. [69,74] analysed ten populations from China including one outgroup population for Slovenia. There are currently 33 COI fragment data entries for *C. ciliata* in the GenBank [75]. Some preliminary results on *C. ciliata* were published in 2020 [76], but this subset of data incorporates only 22 locations, 117 individuals, and a short fragment (546 bp) of the COI gene. Further, Besedina et al. [56] analysed 20 individuals of three populations from Krasnodar (Russia) using RAPD-PCR markers and revealed high DNA polymorphism. However, their main conclusion was that there is no genetic difference between the studied populations.

Several studies used a fragment that was longer (>1100 bp) than the barcode fragment of COI. Some examples of this method include the plant bug *Adelphocoris fasciaticollis* Reuter, 1903 (Hemiptera: Miridae) [77], hoverflies (*Merodon* sp., Diptera: Syrphidae) [78], *Anopheles darlingi* Root, 1926 (Diptera: Culicidae) [79], *Scarabaeus* (Coleoptera: Scarabaeidae) [80], and *Pyllonorycter platani* (Staudinger, 1870 (Lepidoptera: Gracillariidae) [7] to reveal the population genetic structure of the investigated insect taxa. Forensic studies use a longer fragment of COI to identify the Diptera species as well [81].

Our aims were (i) to reveal the genetic structure of *Corythucha ciliata* both in its native and invaded area, (ii) to explore the species’ phylogeographic pattern across three continents (Europe, Asia, and North America), (iii) to revisit the possible introduction events of the species, and (iv) to reanalyse our data with the already published datasets.

## 2. Materials and Methods

### 2.1. Sampling and Molecular Methods

**Figure 1:**
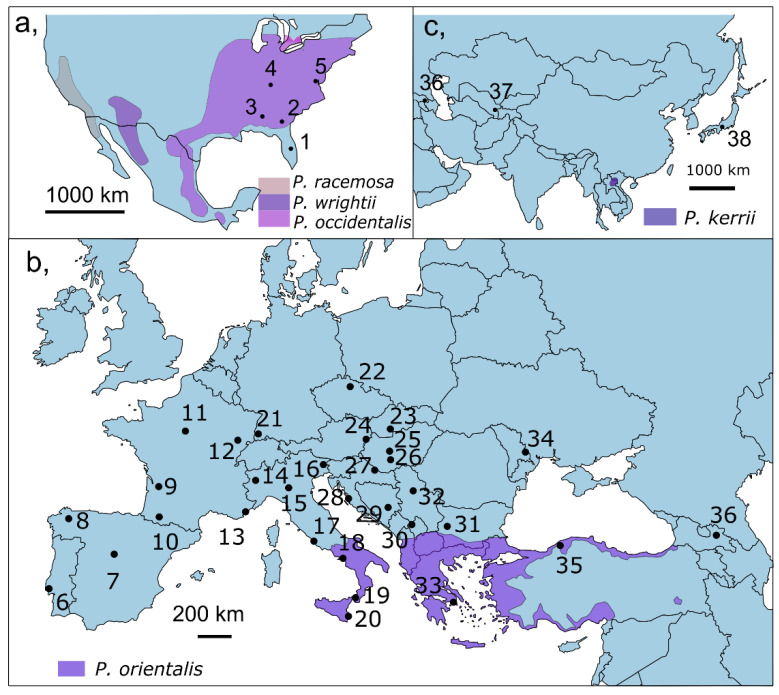
The sampling locations (1–38) of *Corythucha cilata* and the native range of *Platanus* spp. in (**a**) North America, (**b**) Europe, and (**c**) Asia. The native range of *Platanus racemosa* Nutt. ex Audubon, *P. wrightii* S. Wats., *P. occidentalis*, *P. orientalis* and *P. kerrii* Gagnep. are presented based on the map published by Feng et al. [82].

We collected nymphs and imagos from 38 populations of *C. ciliata* from Europe, Central Asia, Japan and North America and one Hungarian population of *Corythucha arcuata* (Say, 1832) (Figure 1, Table S1). All samples were stored in 96% ethanol at 4 °C. DNA was extracted from entire bodies using GenElute Mammalian Genomic DNA Miniprep Kit (Sigma-Aldrich), following the manufacturer protocol. Eluted DNA was stored at −20 °C.

A 1356 bp long region of the COI gene was amplified for 327 individuals by using Pat (5′-TCC AAT GCA CTA ATC TGC CAT ATT A-3′), and LCO1490-J-1514 (5′-GGT CAA ATC ATA AAG ATA TTG G-3′) primers [83,84]. PCR conditions included an initial denaturation step at 94 °C for 2 min, followed by 34 cycles at 94 °C for 30 s, 46 °C for 1 min, and 72 °C for 1 min 30 s with a final extension step that lasted 10 min at 72 °C.

Sequences were generated at the Eurofin’s Laboratory (Ebersberg, Germany). All sequences are available at NCBI GenBank (accession numbers OM033605-621).

### 2.2. Data Analysis

Three hundred and twenty-seven individuals were used for mitochondrial DNA (COI) analyses (Table S1). Sequences were visualized using FinchTV 1.4.0 [85] and then aligned using ClustalX [86]. After haplotypes were identified, those represented by only a single individual were verified by additional sequencing of an independent amplicon. *Corythucha arcuata* (Say, 1832) sequence (OM033622) was used as an outgroup. Genetic distances were estimated using the Kimura 2-parameter and computations were completed in MEGA 5.02 [87].

### 2.3. Phylogenetic Analyses

We applied jModeltest 2.1.2 [88,89] to select the best model of nucleotide substitution with Akaike Information Criterion (AIC) [90]. Maximum likelihood (ML) analysis was performed under GTR+I model with MEGA 5.02. The level of support for individual nodes was evaluated by bootstrapping with 5000 replicates.

Population structure: Patterns of molecular diversity based on the mtDNA sequences between and within populations were assessed by estimating nucleotide diversity (π) [91], transition/transversion ratio, and haplotype diversity (h) [92,93] using the software Arlequin version 3.5.1.2 [94].

Genetic distances between groups (continents; natural-invaded area) and within groups were estimated using the Kimura 2-parameter and computations were completed in MEGA 5.02 [87].

Demographical expansion: Population dynamics analyses were performed on different geographical scales: overall dataset, between natural and invaded area, within natural and within invaded area, between continents, and within continents, with special emphasis on European populations. Arlequin 3.5.1.2 with 10,000 permutations [94] was used for the estimation of Tajima’s D statistics [95] and Fu’s Fs [96]. With small sample sizes (as in the case of 546 bp dataset: <60 individuals); we also used DnaSp 5.10 [97] to estimate R2 [98].

Phylogeographical analysis: Spatial analysis of molecular variance (SAMOVA) was performed using SAMOVA v1.0 [99]. The program was run 1023 iterations. K values were tested, starting from two until the value for which FCT reached a plateau [100]. In addition, alternative groups (e.g., natural and invaded area) were tested with Analysis of Molecular Variance (AMOVA) [101,102,103] with Arlequin 3.5.1.2 [94]. The statistical significance of variance components in AMOVA was tested with 1000 permutations. Statistical parsimony network (SP) [104] was constructed with TCS 1.2.1 [105] and edited using tcsBU [106].

QGIS 2.18.11 [107] was used to project haplotype distributions and frequencies onto maps. Annotations on the maps, phylogenetic trees, and haplotype networks were edited using Inkscape 1.0.2-2 [108].

## 3. Results

### 3.1. Long Fragments of the COI Gene

**Figure 2:**
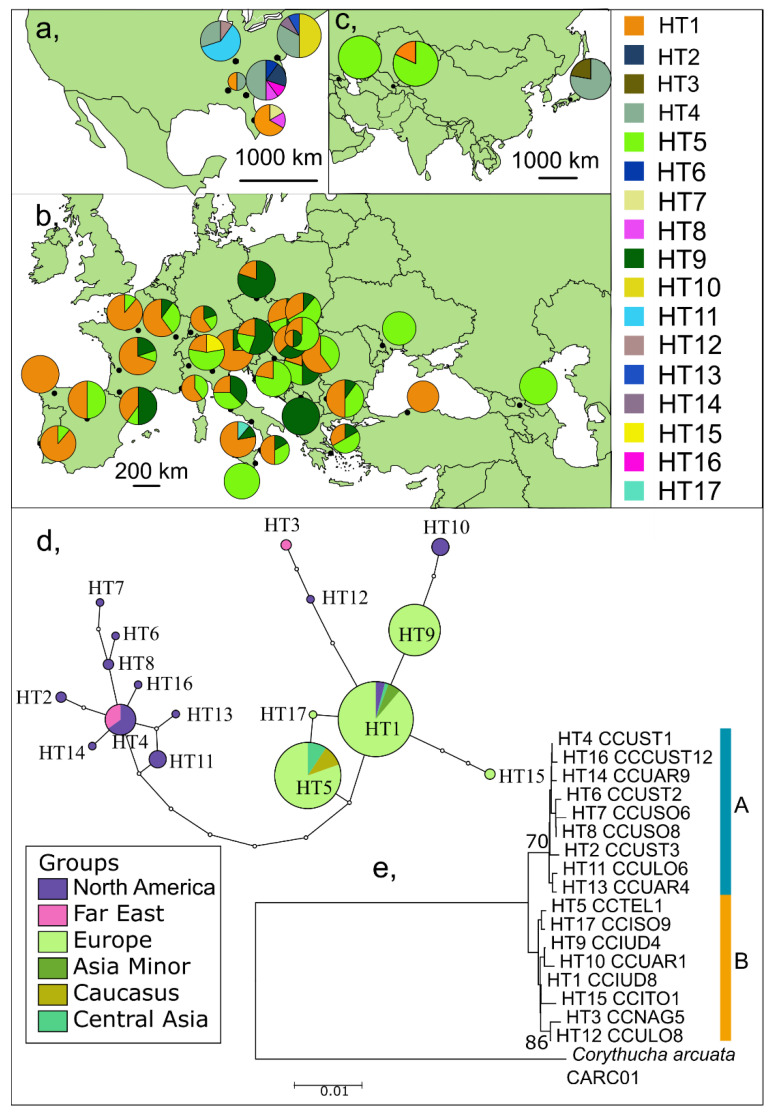
Distribution and phylogenetic relationship of *Corythucha ciliata* haplotypes on the long fragment of COI: (**a**) in North America, (**b**) in Europe, and (**c**) in Asia; (**d**) statistical parsimony networks for all haplotypes (empty circles indicate missing or theoretical haplotypes); and (**e**) ML consensus tree of all COI haplotypes. Bootstrap support values expressed in percentages are indicated near the nodes (>60%).

Seventeen haplotypes were detected on the 1356 bp long fragment of the COI gene from 327 individuals from 38 localities (Figure 2, Table S1). The variable sites numbered 26 (1.92%). Approximately half of them were located on the barcoding part of the gene. Haplotypes were differentiated from each other by 1–10 polymorphic sites.

The haplotypes formed two haplogroups (A and B), which were separated by at least seven mutation steps (Figure 2). The 14 intermediate haplotypes were not present in our data set. The topology of the phylogenetic tree was similar to the haplotype network. Haplogroup A includes nine haplotypes from North America (HT2, 4, 6–8, 11, 13–14, and 16) and one haplotype from Japan (HT4), while haplogroup B includes only three haplotypes from North America (HT1, 10, and 12), five from Europe (HT1, 5, 9, 15, and 17), two from Western and Central Asia (HT1 and 5), and one from Japan (HT3). The most abundant haplotypes are HT1 (38.23% of the total dataset), HT5 (29.66%), and HT9 (17.74%). HT5 and HT9 were only detected from Europe and Central Asia. HT3 is unique from Japan. Most haplotypes were detected from North America only (HT2, HT6–8, HT10–14, and HT16; six of these are singletons). HT4 (6.12%) was found both in Japan and North America. HT15 and HT17 are unique haplotypes from Europe. The average sequence divergence between the haplotypes of the SLB was 0.07–1.04%, much lower than the interspecific divergence between *C. arcuata* and *C. ciliata* 8.49–8.93%.

The genetic distance between populations was 0.00–0.65%; within populations, 0.00–0.52%; and the overall mean distance (TOTAL DATASET) was 0.20%. Overall, haplotype diversity (h) was 0.73, and nucleotide diversity (π) was 0.20% (Table 1).

#### 3.1.1. Genetic Diversity and Structure in the Native Range—North America

Altogether 12 haplotypes were detected among the sequences of the 40 specimens collected in North America (five sampling locations). Ten of these haplotypes (HT2, HT6–8, HT10–14, and HT16) were unique. HT4 was the most common (32.50%) and was found in all populations except Orlando (Figure 2, Table S1). This haplotype was also found in Japan. HT1 and HT8 revealed two populations. All the other haplotypes were detected at single locations. Both haplogroups A and B were represented in this continent (Figure 2). Haplotype diversity (h) was 0.85, and nucleotide diversity (π) was 0.36% (Table 1).

Neutrality test results showed that neither Tajima’s D nor Fu’s Fs values were significant. Mismatch distributions showed multimodal (SSD = 0.041) waves, associated with a constant population size [109,110].

#### 3.1.2. Genetic Diversity and Structure in the Invaded Range

Average sequence divergence between invaded and natural groups (0.46%) was higher than at the intrapopulation level (invaded: 0.12%; natural: 0.35%). The genetic distance within the natural group was nearly three times higher (0.35%) than within the invaded group (0.12%).

Fu’s Fs and Tajima’s D values were not significant. Mismatch distribution (SSD = 0.020) shows a multimodal shape, which suggests a constant population size [109,110].

##### Europe

Five haplotypes were detected among the sequences of the 250 specimens collected in Europe (29 locations). Two of these were common (HT1 44.40% and HT5 31.20%) while HT9 (23.20%), HT15 (0.80%), and HT17 (0.40%) were unique for Europe. HT9 was common in the populations from Central Europe and the Balkan Peninsula. Europe is represented in haplogroup B only (Figure 2). Haplotype diversity (h) was 0.65, and nucleotide diversity (π) was much lower than in North America 0.09% (Table 1). Intrapopulation divergence was 0.10% within the European group.

Mismatch distributions show a slightly unimodal (SSD = 0.015) shape for the European dataset. Unimodal distributions are generally associated with a sudden/recent population expansion or bottleneck [109,110].

##### Asia

Four haplotypes were observed among the 37 specimens collected in Asia (four locations) with one being (HT3) unique to the continent. The population from Japan differs from the other Asian populations unambiguously because HT3 and HT4 were only observed there, while no other Asian or European haplotype was detected there. HT1 (24.32%) and HT5 (51.35%), common in Europe, were found from Asia Minor, the Caucasus and Central Asia, and are included in Haplogroup B. Haplotypes of the Japanese population are present in both haplogroups. Diversity indices are slightly lower than the North American values (h = 0.66, π = 0.24%).

The results of the neutrality tests (Tajima’s D, Fu’s Fs, and mismatch distribution) did not provide significant values.

Intrapopulation divergence was two times higher in the Asian (0.22%) group than in the European (0.10%). The Asian group was better differentiated (due to the population from Japan) than the European group.

The FCT values reached a plateau at K = 4 (FCT = 0.702) by SAMOVA, but the four groups did not consist with the geographic distribution.

Results of AMOVA demonstrated that the largest variation (44.64%) occurs among natural and invaded groups (Table 2), while 31.08% of variation occur among populations within groups and 24.28% within populations.

### 3.2. Short (Barcoding) Fragments of the COI Gene including Already Published Data

**Figure 3:**
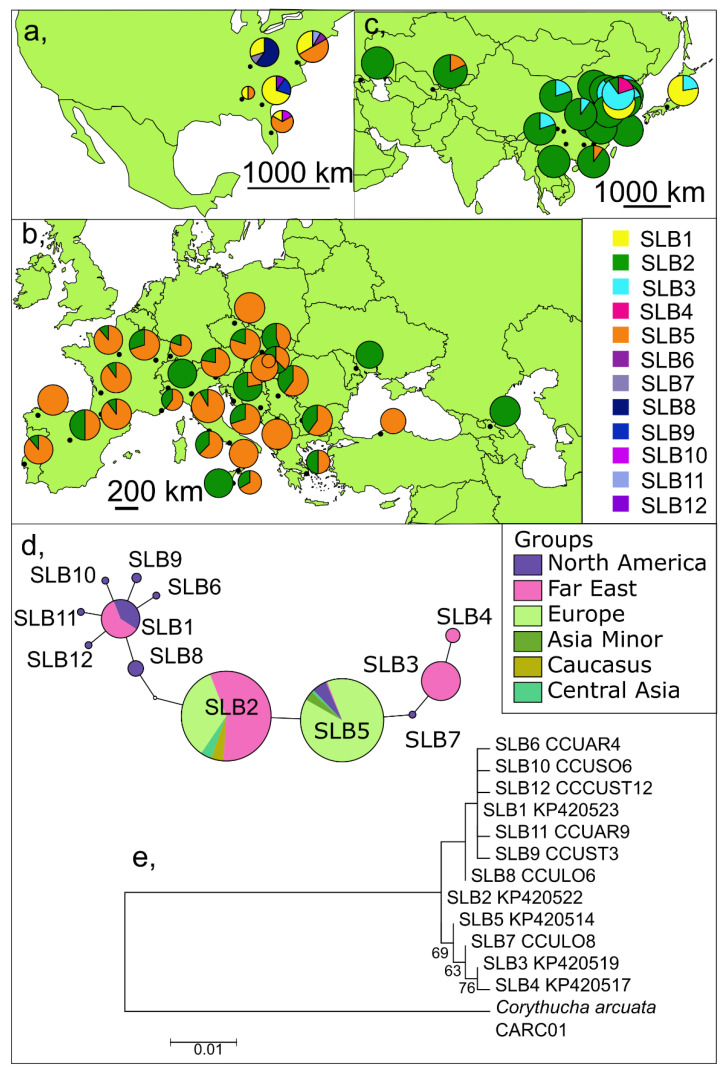
Distribution phylogenetic relationship of *Corythucha ciliata* mitochondrial haplotypes on the barcoding fragment of COI: (**a**) in North America, (**b**) in Europe and (**c**) in Asia; (**d**) Statistical parsimony networks for all haplotypes (empty circles indicate missing or theoretical haplotypes); and (**e**) ML consensus tree of all COI haplotypes. Bootstrap support values expressed in percentages are indicated near the nodes (>60%).

Our data (327 individuals, 38 localities) were supplemented by the results of Yang et al. [74] (190 individuals, 19 localities). The consolidated dataset (517 individuals, 57 localities) contains, altogether, twelve haplotypes on the 546bp long barcoding fragment of the COI gene (Figure 3, Table 3, and Table S1). Yang et al. [74] has described five of these haplotypes; the remaining seven are new. The number of variable sites was 11 in this case (2.01%). The pairwise genetic distances between the haplotypes were 0.18–1.48%.

The haplotype SLB2 was detected in 43.52% of the individuals across the entire invaded area, but it could not be confirmed in the native area. The other common haplotype was SLB5 (37.33%), which was present across all continents. In our samples from Europe (SLB2; SLB5), Asia Minor (SLB5), Caucasus (SLB2), and Central Asia, (SLB2, SLB5) we detected only two haplotypes with various patterns. There were only two, albeit different, haplotypes from Japan (SLB1; SLB3). Yang et al. [74] detected five haplotypes from China (SLB1–5), where only SLB4 was unique. We revealed altogether eight haplotypes from North America (SLB1, SLB5, SLB6–12)—all of the later ones were unique. Neither the ML tree nor the haplotype network supports the existence of well-defined haplogroups on the barcode fragment of COI (Figure 3). We observed a moderate value of the haplotype diversity indices and a low value of the nucleotide diversity in the short fragment of COI (h = 0.66, π = 0.26%) (Table 3).

None of the neutrality tests (including the Tajima’s D, Fu’s Fs indices, and the mismatch distribution) provided significant results.

The FCT values reached a plateau at K = 8 (FCT = 0.652) by SAMOVA, but the set of the eight groups did not consist with the geographic distribution.

AMOVA analysis detected the largest variation (57.47%) among natural and invaded groups (Table 4), while only 15.97% of variation occurs among populations and 26.56% within populations. The fixation index among groups was more than 1.5 times higher than among populations within groups (FCT = 0.575, *p* < 0.01; FSC = 0.376, *p* < 0.01), indicating that there may be factors limiting the gene flow among regions.

#### 3.2.1. Invaded Versus Natural Range

##### Invaded Area

Altogether five haplotypes were observed in the invaded area, where SLB2 and SLB5 were the two most common haplotypes (47.17% and 38.16%). SLB2-SLB4 were detected only from the invaded area.

In the invaded area we detected moderate haplotype diversity with low nucleotide diversity indices (h = 0.62, π = 0.21%). Haplotype diversities were moderate and nucleotide diversities were low in both the Far East (h = 0.53, π = 0.32%) samples and in the European samples (h = 0.42, π = 0.08%).

We observed 0.51% average sequence divergence between invaded and natural populations. Within-group divergence of the invaded area was approximately half (0.21%) of the natural range of within-group divergence (0.40%). Sequence divergence was 0.30% within the Asian group and 0.08% within the European group, while the sequence divergence between Asia and Europe was 0.25%.

The neutrality tests (Tajima’s D, Fu’s Fs, mismatch distribution, and R2) usually were not significant in the most of investigated groups (invaded, Europe, Asia, Far East, etc.) except the invaded group, where the mismatch distribution (SSD = 0.019) had an unimodal shape. This is a common finding in populations that experienced recent population expansion or bottleneck [109,110].

The question of genetic diversity and the term of the invasion is interesting because approximately 60 years have passed since the invasion began in Europe. Regardless, we revealed only two haplotypes (257 individuals; h = 0.42; π = 0.08%) in Europe. Only 20 years have passed since the invasion began in the Far East, yet we revealed five haplotypes (199 individual; h = 0.53, π = 0.32) there.

##### Native Range

Nine haplotypes were detected in the native range where SLB1 (40%), SLB5 (27.5%), and SLB8 (15%) were the most common and SLB6–12 were unique. From North America, we observed high haplotype diversity with low nucleotide diversity (h = 0.76, π = 0.39%) and revealed average sequence divergence (0.40%) that was nearly two times higher than in the invaded area (0.21%). Fu’s Fs and Tajima’s D values were not significant. Mismatch distribution (SSD = 0.035) shows multimodal shape, which is usually associated with constant population size [109,110].

## 4. Discussion

### 4.1. Genetic Diversity of SYCAMORE Lace Bug

We detected moderate haplotype diversity (17 HTs) on the long (1356 bp) fragment of the COI gene in the *Corythucha ciliata* populations. Interspecific divergence of the COI gene in the plant bugs (Miridae) was reported as 6.30% [111]. Park et al. [112] detected more than 3% interspecific divergence for lace bugs (Tingidae), to which *C. ciliata* belongs. The interspecific divergence values between *C. arcuata* and *C. ciliata* varies 8.49–8.93% in our study.

Intraspecific distances for other Heteropteran species were reported 0–7.72% (mean distance 0.74%) [112], and for *Apolygus* species (Miridae) 0.40% [111]. In our study, the overall mean distance was 0.20% and the distance between populations was 0.00–0.65%. Jung et al. [111] revealed that in some cases the average interspecific genetic distance between closely related species was 32 times higher than the average intraspecific distance (e.g., genus *Scolopocelis*). In our study, we also detected 44 times higher interspecific divergence. COI sequences of *C. ciliata* showed higher genetic differentiation than avocado lace bug (*P. perseae*), where altogether nine haplotypes from 469 individuals with 16 polymorphic sites were found [113]. The haplotype diversity is relatively high (h = 0.73) on the 1356 bp long fragment of COI, but with low nucleotide diversity (π = 0.20%), which predicts a population bottleneck followed by rapid population growth and accumulation of mutations [114]. While we found slightly higher values of all diversity indices in the native group than in the introduced one, we found also high haplotype diversity with low nucleotide diversity values (h = 0.85; π = 0.36%), which also supports the above conclusion [114]. Furthermore, these results show that we have incomplete information about the past and current distribution and about the genetic structure of SLB in North America. This is reflected in discontinuous distribution records (e.g., the occurrence in the eastern part of the Rocky Mountains) [8] as well. In the invaded regions, we found relative high haplotype diversity with low nucleotide diversity (Europe h = 0.65, π = 0.09%; and Asia h = 0.66, π = 0.24%), which also suggests a population bottleneck followed by rapid population growth and accumulation mutations [114]. Several authors [1,7,74,115] report the loss of genetic diversity for invasive species under the process of biological invasion.

The genetic structure (Figure 2) together with the known invasion history of the SLB [9,10,11,12,13,14,15,16,17,18,19,20,21,22,23,24,25,26,27,28,29,30,31,32,33,34,35,36,37,38,39,40,41,42,43,44] suggest that there was only a single introduction event in Europe.

### 4.2. Long Versus Short (Barcoding) Fragments of the COI

Several papers have analysed the applicability of COI fragments of different lengths and locations on the mtDNA. Roe & Sperling [114] suggest the use of a longer COI fragment in pilot studies on any taxon. Maggioni et al. [115] experienced that COI-3′ regions were slightly more variable than 5′. Therefore, they recommend using this part of the mtDNA to assess the intraspecific geographic structure of Odonata species.

We detected significantly higher values in most of the diversity indices (No, S, ts, tv, and h) values on the longer fragment than on the barcode fragment. Our study could not reveal all possible links and connections in the invaded areas of *Corythucha ciliata*. New populations need to be included in future analyses, particularly in Asia.

## Figures and Tables

**Table 1:** Summary of genetic diversity indices for the long fragment of the COI gene: (n) number of individuals sampled; (No) number of haplotypes; (Nex) number of exclusive haplotypes; (S) number of polymorphic sites; (h) haplotype diversity; (π) nucleotide diversity; (n.r.) not relevant; (E) Europe; (A) Asia; and (NA) North America.

Group	n	No.	Nex	S	ts/tv	h ± SD	π (%) ± SD
Invaded	287	7	4	16	14/2	0.6717 ± 0.0124	0.1196 ± 0.0785
E	250	5	3	6	6/0	0.6542 ± 0.0123	0.0937 ± 0.0655
A	37	4	1	12	10/2	0.6562 ± 0.0555	0.2401 ± 0.1406
Native/NA	40	12	10	21	20/1	0.8462 ± 0.0378	0.3630 ± 0.2005
**Total**	327	17	n.r.	26	25/2	0.7320 ± 0.0136	0.1945 ± 0.1152

**Table 2:** Analysis of molecular variance (AMOVA) for the natural and invaded groups of *C. ciliata*, long COI fragment (*** *p* < 0.001).

Groups	Source of Variation	var%	Fixation Indices
Natural	Among groups	Va = 44.64	FCT = 0.446 ***
Invaded	Among populations within groups	Vb = 31.08	FSC = 0.561 ***
	Within populations	Vc = 24.28	FST = 0.757 ***

**Table 3:** Summary of genetic diversity indices for the barcoding fragment of the COI gene: (n) number of individuals sampled; (No) number of haplotypes; (Nex) number of exclusive haplotypes; (S) number of polymorphic sites; (h) haplotype diversity; (π) nucleotide diversity; (n.r.) not relevant; (E) Europe; (A) Asia; and (NA) North America.

Group	n	No.	Nex	S	ts/tv	h ± SD	π (%) ± SD
Invaded	477	5	3	7	6/1	0.6232 ± 0.0125	0.2120 ± 0.1517
E	250	2	0	1	1/0	0.4310 ± 0.0222	0.0789 ± 0.0791
A	227	7	2	7	6/1	0.5368 ± 0.0331	0.2996 ± 0.1964
Native/NA	40	9	7	10	10/1	0.7551 ± 0.0456	0.3933 ± 0.2479
**Total**	517	12	n.r.	11	11/1	0.6600 ± 0.0122	0.2552 ± 0.1737

**Table 4:** Analysis of molecular variance (AMOVA) for two groups (natural and invaded area) of *C. ciliata*, barcoding fragment of the COI (*** *p* < 0.001).

Groups	Source of Variation	var%	Fixation Indices
Natural	Among groups	Va = 57.47	FCT = 0.575 ***
Invaded	Among populations within groups	Vb = 15.97	FSC = 0.376 ***
	Within populations	Vc = 26.56	FST = 0.734 ***

## Data Availability

The following data are available in Supplementary Materials, Table S1: GenBank accession numbers for nucleotide sequences generated for this study (accession numbers OM033605-621). [dataset] Yang, W.Y.; Tang, X.T.; Ju, R.T.; Zhang, Y.; Du, Y.Z. 2017. The Population Genetic Structure of *Corythucha ciliata* (Say) (Hemiptera: Tingidae) Provides Insights into Its Distribution and Invasiveness. *Sci. Rep.* **2017**, *7*, 635; (GenBank accession numbers KP420514-523).

## Supplementary Materials

The following supporting information can be downloaded at https://www.mdpi.com/article/10.3390/insects13020123/s1: Table S1, COI haplotypes distributions.
