# Supplementary material for: Longer mtDNA Fragments Provide a Better Insight into the Genetic Diversity of the Sycamore Lace Bug, Corythucha ciliata (Say, 1832) (Tingidae, Hemiptera), Both in Its Native and Invaded Areas
Source: Insects. 2022 Jan 25;13(2):123. doi: 10.3390/insects13020123 (PMC8875602; doi:10.3390/insects13020123)
Supplement: Supplementary file 1 [file insects-13-00123-s001.zip › insects-1556529-supplementary.pdf]

**Table S1.** Origin, number of haplotypes of investigated *Corythucha ciliata* populations. N: the number of individuals.

| | Country | Location | Year | Lat. | Long. | N. | Haplotypes on the 1356 bp Fragment of COI | Haplotypes on the Barcoding Fragment of COI |
|---|---|---|---|---|---|---|---|---|
| 1 | USA | Orlando (FL) | 2009 | 28.50 | -81.44 | 6 | HT1 (4); HT7 (1); HT8 (1) | SLB1 (1); SLB5 (4); SLB10 (1) |
| 2 | USA | Tifton (GA) | 2009 | 31.46 | -83.50 | 10 | HT2 (2); HT4 (5); HT6 (1); HT8 (1); HT16 (1) | SLB1 (7); SLB9 (2); SLB12 (1) |
| 3 | USA | Montgomery (AL) | 2009 | 32.37 | -86.26 | 2 | HT1 (1); HT4 (1) | SLB1 (1); SLB5 (1) |
| 4 | USA | Louisville (KY) | 2012 | 38.16 | -85.79 | 10 | HT4 (3); HT11 (6); HT12 (1) | SLB1 (3); SLB7 (1); SLB8 (6) |
| 5 | USA | Arlington (VA) | 2012 | 38.86 | -77.08 | 12 | HT4 (4); HT10 (6); HT13 (1); HT14 (1) | SLB1 (4); SLB5 (6); SLB6 (1); SLB11 (1) |
| 6 | Portugal | Lisabon | 2014 | 38.89 | -9.14 | 9 | HT1 (8); HT5 (1) | SLB2 (1); SLB5 (8) |
| 7 | Spain | Valsain | 2013 | 40.99 | -4.05 | 10 | HT1 (5);HT5 (5) | SLB2 (5); SLB5 (5) |
| 8 | Spain | Lugo | 2012 | 43.01 | -7.56 | 10 | HT1 (10) | SLB5 (10) |
| 9 | France | Branne | 2010 | 45.07 | -0.20 | 10 | HT1 (7); HT5 (1); HT9 (2) | SLB2 (1); SLB5 (9) |
| 10 | France | Coarse | 2010 | 43.64 | -0.20 | 10 | HT1 (4); HT5 (1); HT9 (5) | SLB2 (1); SLB5 (9) |
| 11 | France | Paris | 2011 | 48.87 | 2.32 | 9 | HT1 (8); HT5 (1) | SLB2 (1); SLB5 (8) |
| 12 | France | Le Puy | 2010 | 47.35 | 6.23 | 10 | HT1 (6); HT5 (3); HT9 (1) | SLB2 (3); SLB5 (7) |
| 13 | France | St. Räphael | 2010 | 43.54 | 6.75 | 5 | HT1 (3); HT5 (2) | SLB2 (2); SLB5 (3) |
| 14 | Italy | Torino | 2010 | 45.08 | 7.72 | 9 | HT1 (2); HT5 (5); HT15 (2) | SLB2 (5); SLB5 (4) |
| 15 | Italy | Parma | 2010 | 44.94 | 10.30 | 12 | HT1 (9); HT5 (1); HT9 (2) | SLB2 (1); SLB5 (11) |
| 16 | Italy | Udine | 2011 | 46.07 | 13.24 | 9 | HT1 (2); HT5 (2); HT9 (5) | SLB2 (2); SLB5 (7) |
| 17 | Italy | Ostia | 2011 | 42.00 | 12.27 | 8 | HT1 (2); HT5 (3); HT9 (3) | SLB2 (3); SLB5 (5) |
| 18 | Italy | Salerno | 2011 | 40.68 | 14.78 | 9 | HT1 (7); HT9 (1); HT17 (1) | SLB5 (9) |
| 19 | Italy | Scilla | 2011 | 38.32 | 15.71 | 6 | HT1 (3); HT5 (2); HT9 (1) | SLB2 (2); SLB5 (4) |
| 20 | Italy | Siracusa | 2011 | 37.08 | 15.29 | 9 | HT5 (9) | SLB2 (9) |
| 21 | Germany | Freiburg | 2013 | 48.00 | 7.85 | 5 | HT1 (3); HT5 (1); HT9 (1) | SLB2 (1); SLB5 (4) |
| 22 | Czechia | Jičin | 2020 | 50.45 | 15.38 | 10 | HT1 (2); HT9 (8) | SLB5 (10) |
| 23 | Slovakia | Leva | 2011 | 48.22 | 18.60 | 9 | HT1 (3); HT5 (5); HT9 (1) | SLB2 (5); SLB5 (4) |
| 24 | Hungary | Sopron | 2010 | 47.68 | 16.58 | 10 | HT1 (3); HT5 (2); HT9 (5) | SLB2 (2); SLB5 (8) |
| 26 | Hungary | Szekszárd | 2010 | 46.35 | 18.71 | 8 | HT1 (3); HT5 (5) | SLB2 (5); SLB5 (3) |

| | | | | | | | | |
|---|---|---|---|---|---|---|---|---|
| 27 | Hungary | Sárbogárd | 2011 | 46.90 | 18.61 | 2 | HT1 (1); HT9 (1) | SLB5 (2) |
| 27 | Croatia | Virovitica | 2012 | 45.84 | 17.38 | 8 | HT1 (3); HT9 (5) | SLB5 (8) |
| 28 | Croatia | Zadar | 2012 | 44.12 | 15.23 | 9 | HT1 (2); HT5 (7) | SLB2 (7); SLB5 (2) |
| 29 | Bosnia and Herzegovina | Sarajevo | 2013 | 43.86 | 18.41 | 10 | HT1 (2); HT5 (3); HT9 (5) | SLB2 (3); SLB5 (7) |
| 30 | Kosovo | Pejë | 2013 | 46.66 | 20.29 | 10 | HT9 (10) | SLB5 (10) |
| 31 | Bulgaria | Sofia | 2012 | 42.71 | 23.33 | 10 | HT1 (5); HT5 (4); HT9 (1) | SLB2 (4); SLB5 (6) |
| 32 | Serbia | Belgrad | 2010 | 44.82 | 20.44 | 10 | HT1 (6); HT5 (4) | SLB2 (4); SLB5 (6) |
| 33 | Greece | Olympus | 2017 | 38.19 | 23.78 | 6 | HT1 (2); HT5 (3); HT9 (1) | SLB2 (3); SLB5 (3) |
| 34 | Moldova | Trisapol | 2012 | 46.84 | 29.63 | 8 | HT5 (8) | SLB2 (8) |
| 35 | Turkey | Karabük | 2012 | 41.21 | 32.62 | 7 | HT1 (7) | SLB5 (7) |
| 36 | Georgia | Telavi | 2011 | 41.92 | 45.48 | 10 | HT5 (10) | SLB2 (10) |
| 37 | Uzbekistan | Samarkand | 2011 | 39.65 | 66.96 | 11 | HT1 (2); HT5 (9) | SLB2 (9); SLB5 (2) |
| 38 | Japan | Nagoya | 2011 | 35.15 | 136.92 | 9 | HT3 (2); HT4 (7) | SLB1 (7); SLB3 (2) |
